# Supplementary material for: Attenuated CSF‐1R signalling drives cerebrovascular pathology
Source: EMBO Mol Med. 2020 Dec 22;13(2):e12889. doi: 10.15252/emmm.202012889 (PMC7863388; doi:10.15252/emmm.202012889)

Appendix

Table of Contents

S1. Cell stress pathway results from variant CSF-1R expressing HEK cells.
S2. IHC of control human tissue.

Appendix Figure S1. Variant CSF-1R does not activate cell stress pathways
Native and P824R CSF-1R – transfected HEKs analysed using the RT² Profiler™ PCR Array Human Stress & Toxicity PathwayFinder. *CXCL8* and *DDIT3* were observed to have a significant 4 and 2-fold downregulation respectively in variant-CSF-1R expressing HEKs.

Appendix Figure S2. IHC of control human cortical tissue.
A-C IHC of non-demented human cortical vessels for **(A)** occludin (red), ZO-1 (green), **(B)** claudin-5 (green), CD68 (red), and **(C)** GFAP (green). Scale bars indicate 20 µm **(A, B)** and 50 µm **(C)**.

Appendix Figure S1

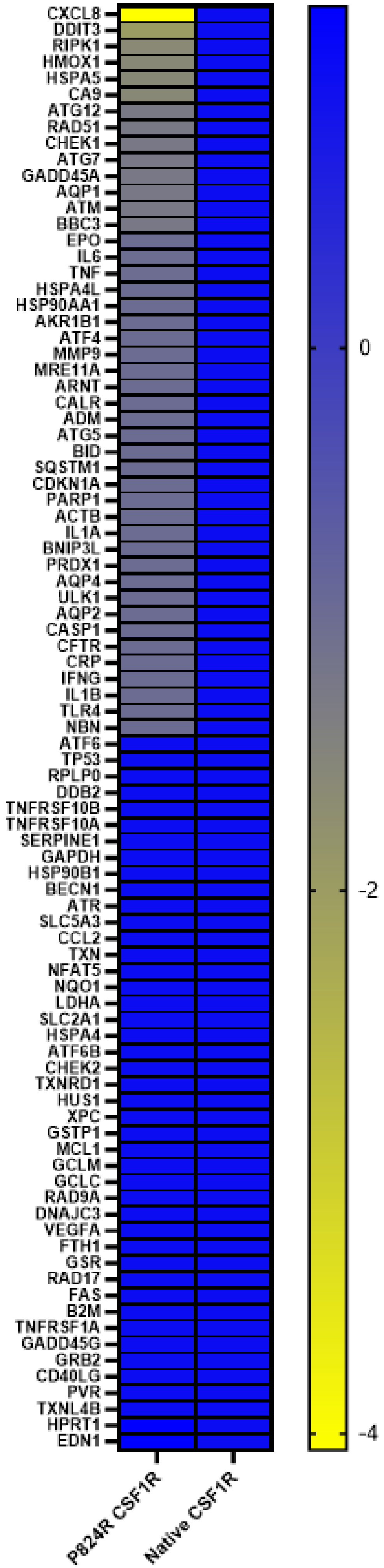

Appendix Figure S2

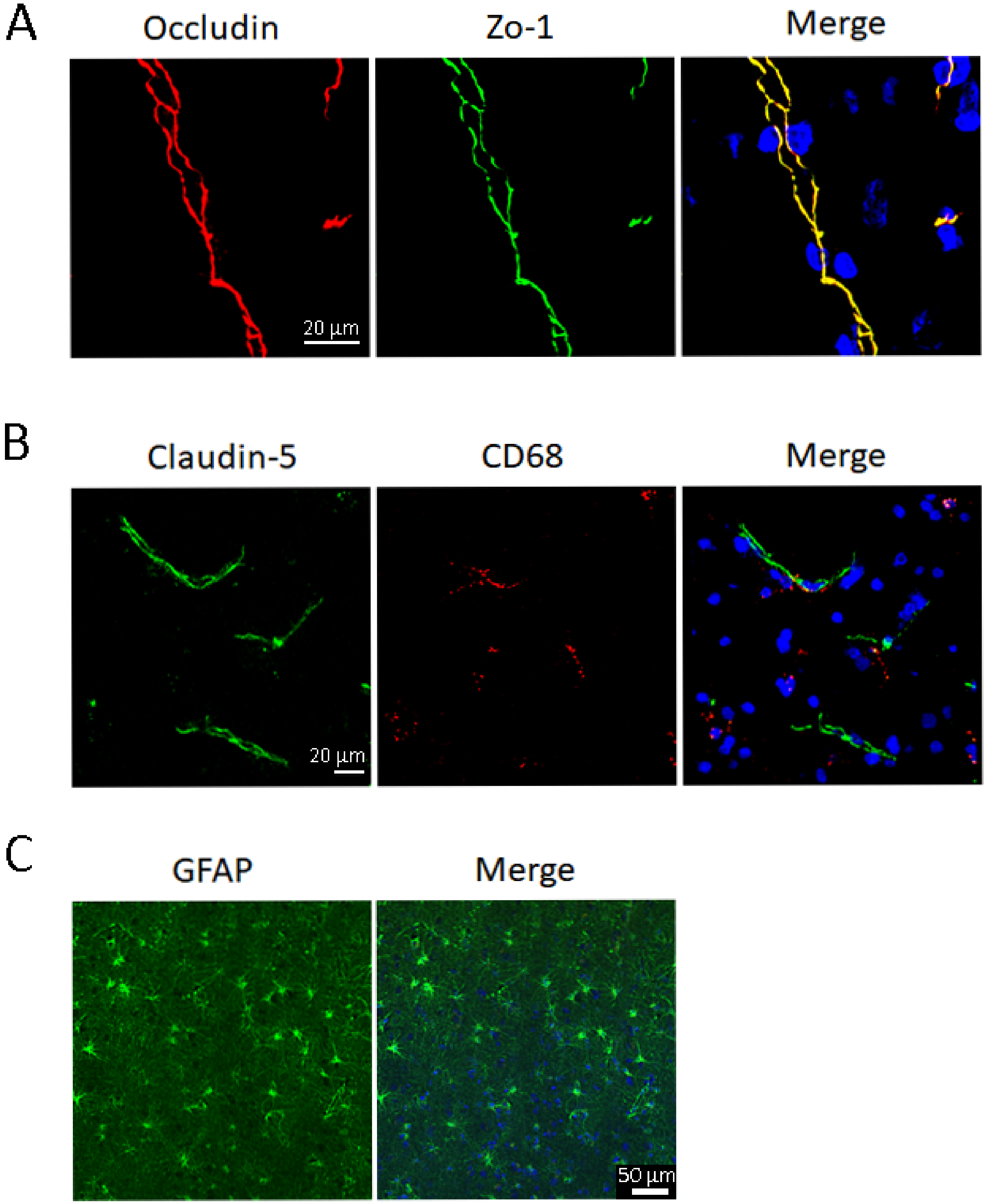

Supplement: Supplementary file 1 — Appendix [file EMMM-13-e12889-s001.pdf]
