# Supplementary material for: Attenuated CSF‐1R signalling drives cerebrovascular pathology
Source: EMBO Mol Med. 2020 Dec 22;13(2):e12889. doi: 10.15252/emmm.202012889 (PMC7863388; doi:10.15252/emmm.202012889)
Supplement: Supplementary file 2 — Expanded View Figures PDF [file EMMM-13-e12889-s002.pdf]

Expanded View Figures

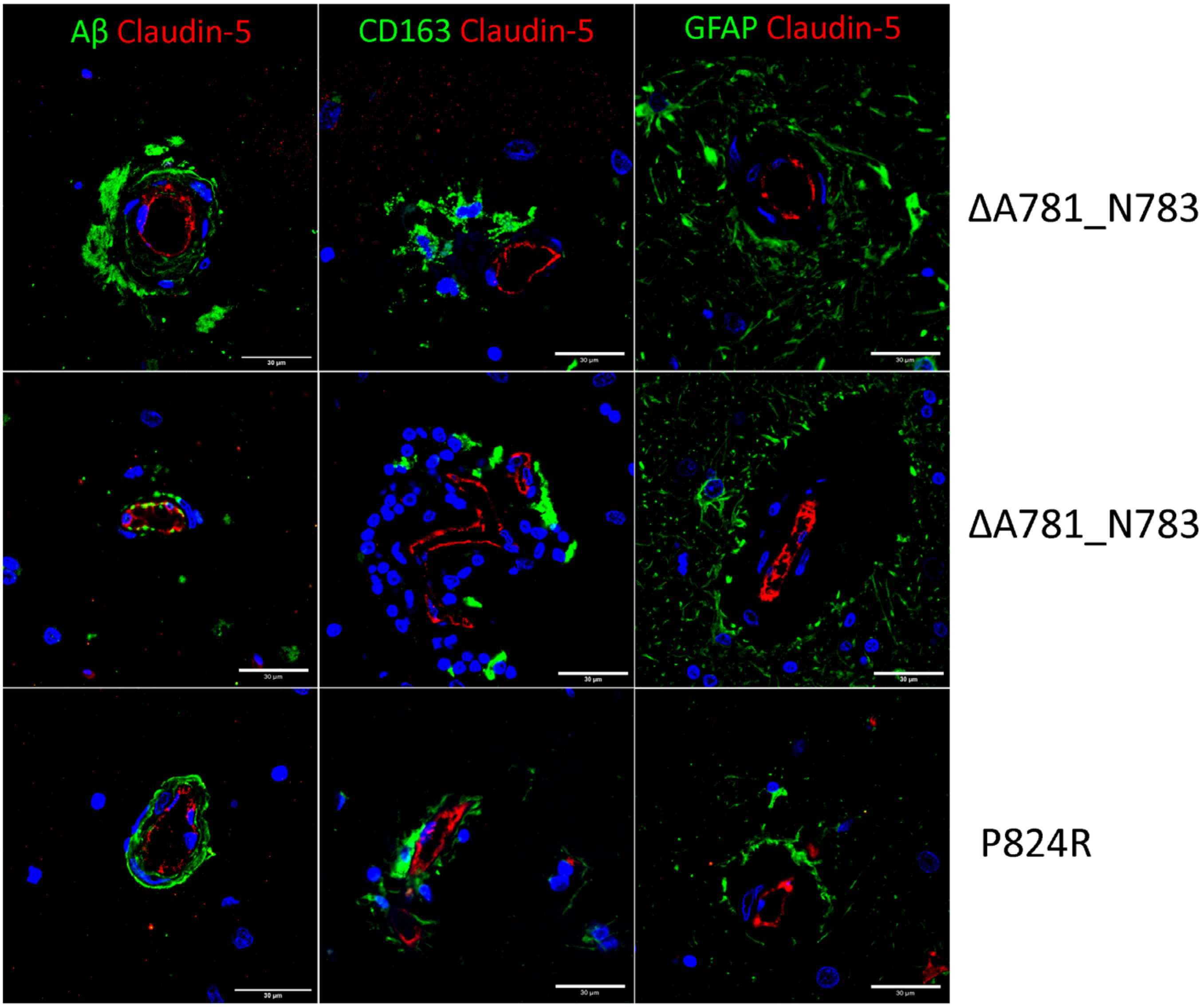

Figure EV1. IHC of individual ALSP brain sections.

IHC of individual ALSP patient post-mortem tissue for amyloid-β (green) and CLD5 (red), GFAP (green) and CLD5 (red), CD163 (green) and CLD5 (red). Note that claudin-5/GFAP images and claudin-5/CD163 images are the same as those shown in Fig 6C and F and are shown here again for ease of comparison. Scale bars indicate 30 μm.

 © 2021 The Authors

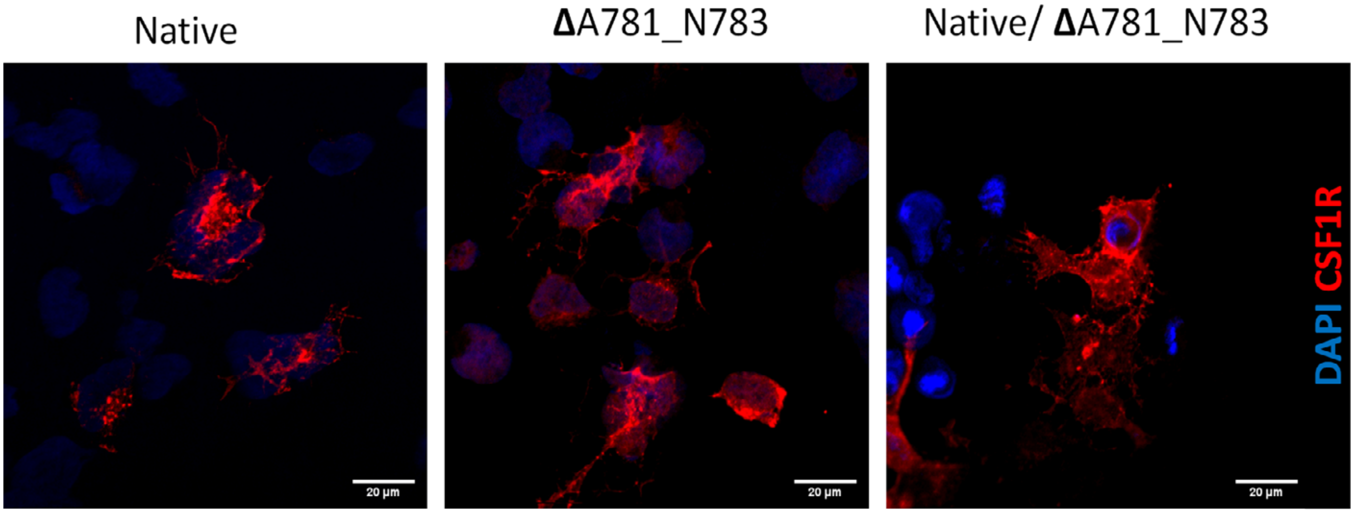

Figure EV2. Immunocytochemistry of CSF-1R expressing HEK293 cells.

ICC for CSF-1R (red) and DAPI (blue) showing membrane localisation of both native and variant CSF-1R. Scale bars indicate 30 μm.

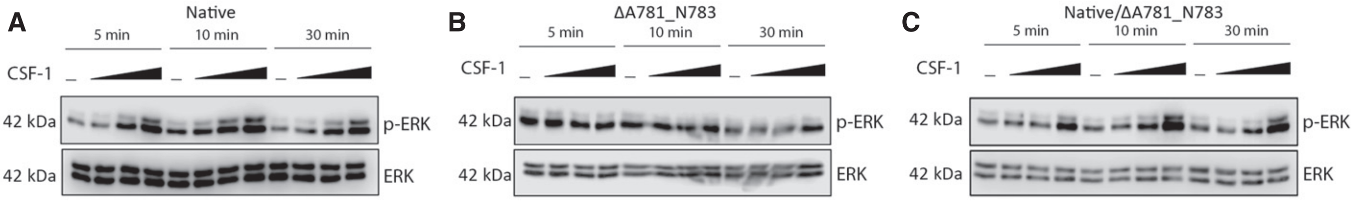

Figure EV3. Restoration of variant CSF-1R levels does not restore function.

A–C qPCR of wild type (blue) or *Csf1r*$^{+/-}$ (red) endothelial cells treated with control, *Csf1r*$^{+/+}$ MCM or *Csf1r*$^{+/-}$ MCM. Statistical analyses of inter-genotype changes. (*$P < 0.05$, **$P < 0.005$, ***$P < 0.0008$, ****$P < 0.0001$. Scatter plots represent technical replicates of $n = 2$ independent primary cell isolations and microglia conditionings, two-way ANOVA with multiple comparisons and Sidak's post-test, error bars indicate SEM).Western blot for phosphorylated and total ERK in HEK293 cells transfected with (A) native CSF-1R, (B) ΔA781_N783 CSF-1R or (C) both native and ΔA781_N783 CSF-1R. Cells were treated with MG132 for 1 h, followed by treatment with CSF1 for 10 min. Horizontal line indicates untreated cells, with increasing concentrations 10, 50 and 100 ng/ml CSF1.

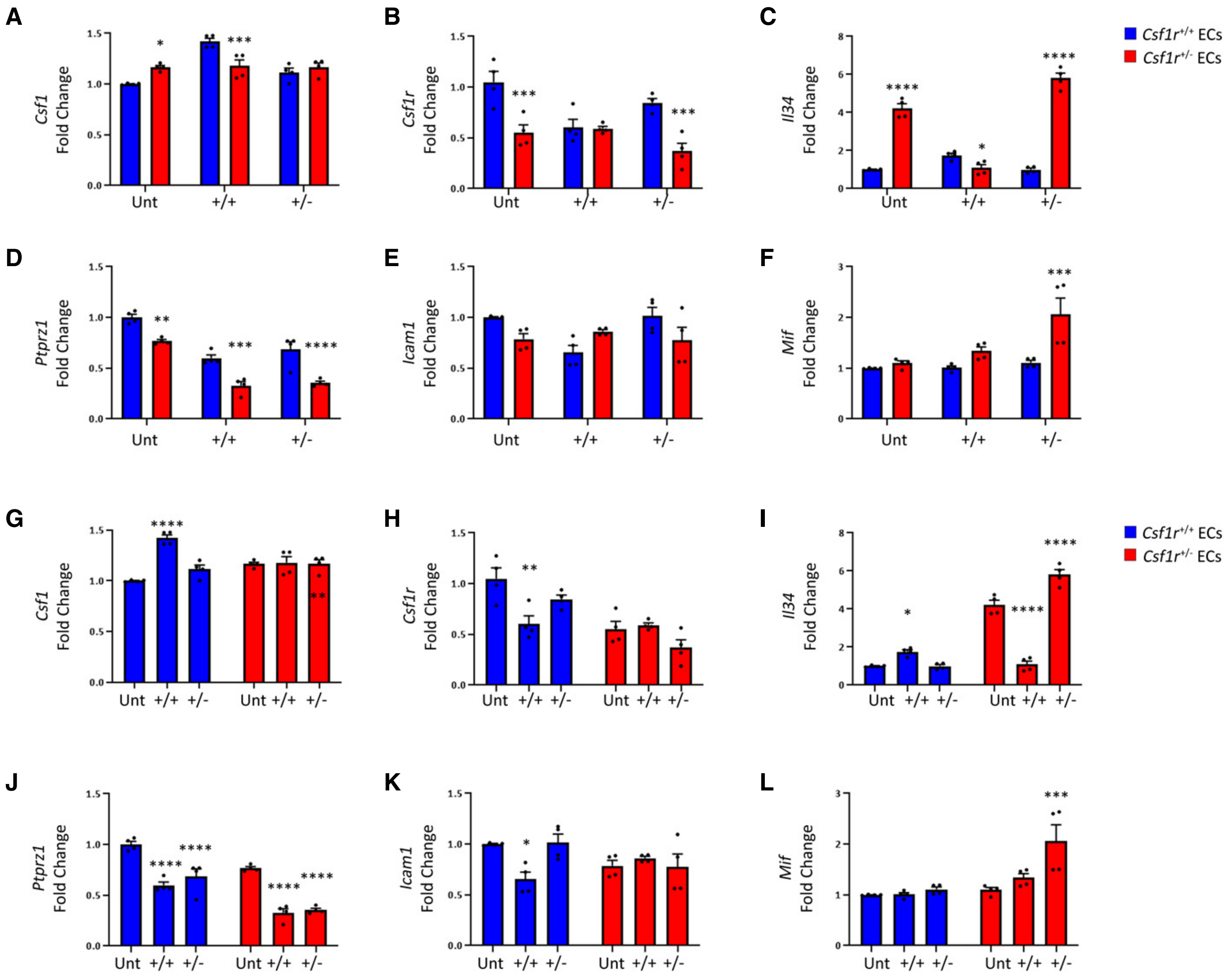

Figure EV4. Transcriptional changes in the CSF-1R pathway of MCM-treated ECs.

A–F qPCR of wild type (blue) or *Csf1r*$^{+/-}$ (red) endothelial cells treated with control, *Csf1r*$^{+/+}$ MCM or *Csf1r*$^{+/-}$ MCM. Statistical analyses of inter-genotype changes. (*$P < 0.05$, **$P < 0.005$, ***$P < 0.0008$, ****$P < 0.0001$. Scatter plots represent technical replicates of $n = 2$ independent primary cell isolations and microglia conditionings, two-way ANOVA with multiple comparisons and Sidak's post-test, error bars indicate SEM).

G–L qPCR of wild type (blue) or *Csf1r*$^{+/-}$ (red) endothelial cells treated with control, *Csf1r*$^{+/+}$ MCM or *Csf1r*$^{+/-}$ MCM. Statistical analyses of changes relative to respective untreated control. (*$P < 0.05$, **$P < 0.008$, ***$P < 0.0008$, ****$P < 0.0001$. Scatter plots represent technical replicates of $n = 2$ independent primary cell isolations and microglia conditionings, two-way ANOVA with multiple comparisons and Sidak's post-test, error bars indicate SEM).

 © 2021 The Authors

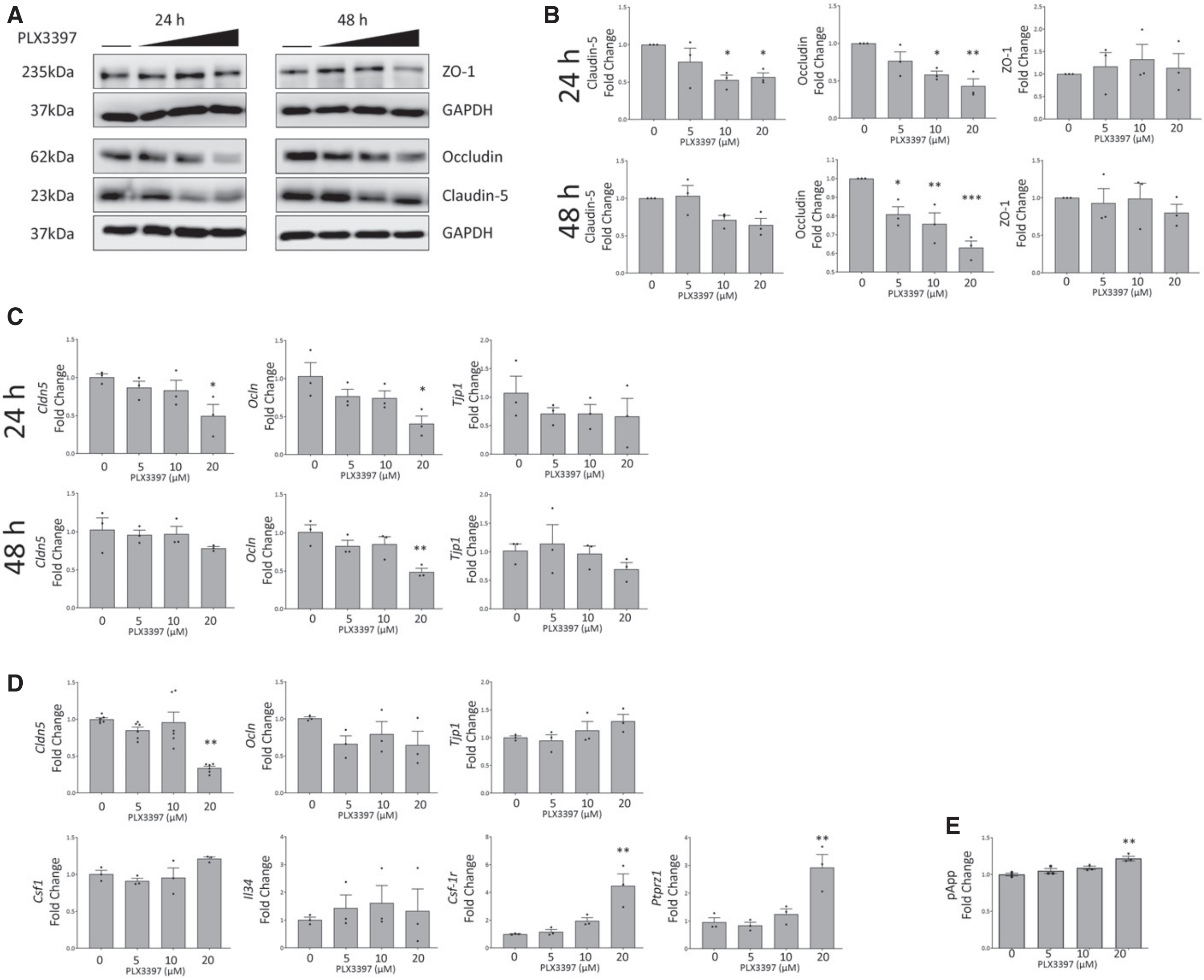

Figure EV5. PLX3397 reduces tight junction expression.

A, B Western blot of lysates from PLX3397 treated b.End3 cells for tight junction proteins ZO-1, Occludin and Claudin-5 (A). The horizontal line indicates untreated cells, with increasing PLX3397 concentrations (5, 10, 20 μM). Corresponding densitometry is given in (B). (One-way ANOVA with Dunnett's post-test for multiple comparisons, *$P < 0.05$, **$P < 0.005$, ***$P < 0.0005$, $n = 3$ independent experiments, error bars indicate SEM)

C Gene expression changes at 24 (top) and 48 (bottom) h in PLX3397 treated b.End3 cells shown by qPCR for *Tjp1*, *Ocln* and *Cldn5* (*$P < 0.05$, **$P < 0.006$, $n = 3$ independent experiments one-way ANOVA with Dunnett's post-test, error bars indicate SEM).

D qPCR analysis of tight junction and CSF-1R pathway gene expression changes at 24 h in PLX3397 treated MBECs (one-way ANOVA with Dunnett's post-test for multiple comparisons, **$P < 0.009$, $n = 3$ independent experiments, error bars indicate SEM).

E FITC-4kDA transwell permeability assay of primary mouse microvascular endothelial cells (MBECs) treated for 24 h with PLX3397 at indicated doses (one-way ANOVA with Dunnett's correction, $n = 3$ technical replicates for flux assay, one-way ANOVA with Dunnett's post-test for multiple comparisons, **$P = 0.0012$, error bars indicate SEM).

© 2021 The Authors
